# The accelerated waning of immunity and reduced effect of booster in patients treated with bDMARD and tsDMARD after SARS-CoV-2 mRNA vaccination

**DOI:** 10.3389/fmed.2023.1049157

**Published:** 2023-02-09

**Authors:** Selma Tobudic, Elisabeth Simader, Thomas Deimel, Jennifer Straub, Felix Kartnig, Leonhard X. Heinz, Peter Mandl, Helmuth Haslacher, Thomas Perkmann, Lisa Schneider, Thomas Nothnagl, Helga Radner, Florian Winkler, Heinz Burgmann, Karin Stiasny, Gottfried Novacek, Walter Reinisch, Daniel Aletaha, Stefan Winkler, Stephan Blüml

**Affiliations:** ^1^Division of Infectious Diseases and Tropical Medicine, Department of Internal Medicine I, Medical University of Vienna, Vienna, Austria; ^2^Division of Rheumatology, Department of Internal Medicine III, Medical University of Vienna, Vienna, Austria; ^3^Department of Laboratory Medicine, Medical University of Vienna, Vienna, Austria; ^4^Department of Second Medical, Lower Austrian Centre for Rheumatology, Korneuburg-Stockerau Hospital, Stockerau, Austria; ^5^Center for Virology, Medical University of Vienna, Vienna, Austria; ^6^Division of Gastroenterology and Hepatology, Department of Internal Medicine III, Medical University of Vienna, Vienna, Austria

**Keywords:** SARS-CoV-2, arthritis, IBD, humoral immune response, immunomodulatory therapy, bDMARD

## Abstract

**Objectives:**

This study aimed to assess the duration of humoral responses after two doses of SARS-CoV-2 mRNA vaccines in patients with inflammatory joint diseases and IBD and booster vaccination compared with healthy controls. It also aimed to analyze factors influencing the quantity and quality of the immune response.

**Methods:**

We enrolled 41 patients with rheumatoid arthritis (RA), 35 with seronegative spondyloarthritis (SpA), and 41 suffering from inflammatory bowel disease (IBD), excluding those receiving B-cell-depleting therapies. We assessed total anti-SARS-CoV-2 spike antibodies (Abs) and neutralizing Ab titers 6 months after two and then after three doses of mRNA vaccines compared with healthy controls. We analyzed the influence of therapies on the humoral response.

**Results:**

Patients receiving biological or targeted synthetic disease-modifying antirheumatic drugs (b/tsDMARDs) showed reduced anti-SARS-CoV-2 S Abs and neutralizing Ab titers compared with HC or patients receiving conventional synthetic (cs)DMARDs 6 months after the first two vaccination doses. Anti-SARS-CoV-2 S titers of patients with b/tsDMARDs declined more rapidly, leading to a significant reduction in the duration of vaccination-induced immunity after two doses of SARS-CoV-2 mRNA vaccines. While 23% of HC and 19% of patients receiving csDMARDs were without detectable neutralizing Abs 6 months after the first two vaccination doses, this number was 62% in patients receiving b/tsDMARDs and 52% in patients receiving a combination of csDMARDs and b/tsDMARDs. Booster vaccination led to increased anti-SARS-CoV-2 S Abs in all HC and patients. However, anti-SARS-CoV-2 S Abs after booster vaccination was diminished in patients receiving b/tsDMARDs, either alone or in combination with csDMARDs compared to HC.

**Conclusion:**

Patients receiving b/tsDMARDs have significantly reduced Abs and neutralizing Ab titers 6 months after mRNA vaccination against SARS-CoV-2. This was due to a faster decline in Ab levels, indicating a significantly reduced duration of vaccination-induced immunity compared with HC or patients receiving csDMARDs. In addition, they display a reduced response to a booster vaccination, warranting earlier booster vaccination strategies in patients under b/tsDMARD therapy, according to their specific Ab levels.

## Introduction

Vaccination against severe acute respiratory syndrome coronavirus type 2 (SARS-CoV-2) is one of the cornerstones in the efforts to curb the disastrous effects of the pandemic and to protect people from severe coronavirus disease 2019 (COVID-19). Although the initial immune response to vaccination against SARS-CoV-2 has been studied in detail (1–6), it is important to investigate the duration of protective immunity to be able to formulate data-driven vaccination strategies. Unfortunately, it has become clear that in the general population, protective immunity against SARS-CoV-2 is not long lasting, as evidenced by decreasing titers and increased breakthrough infection rates over time after initial vaccination (7, 8), as also evidenced by the gradual shortening of recommended intervals for revaccination. People with various immune-mediated diseases need special attention in this regard, as both their underlying diseases and/or the respective treatment regimens possibly alter the response to vaccination. Various immunomodulatory drugs, especially rituximab and mycophenolate, have been shown to severely interfere with the initial response to vaccination. However, disease-modifying antirheumatic drugs (DMARDs) commonly used to treat inflammatory joints, bowel, skin, or other autoimmune diseases were shown to have a more subtle influence on the primary vaccine response (9–15). Given the risk of this group of patients for developing severe COVID-19, understanding the impact of immunomodulatory therapies on immunity over time is of particular clinical relevance. So far, different autoimmune diseases under immunomodulatory therapy and the antibody (Ab) development after SARS-CoV-2 vaccination has been the subject of various studies (16, 17). Yet, studies comparing different autoimmune disease entities and their antibody development after immunization are scarce. To address this lack of information, we chose to match antibody levels of inflammatory arthritis (IA) and inflammatory bowel disease (IBD). The rationale behind the selection of IBD was the similarities in the mode of action of treatment for both illnesses. Many bDMARDs are used in IBD and IA either, especially tumor necrosis factor (TNF) inhibitors or Janus kinase (JAK) inhibitors. In this report, we analyzed antibody development against the SARS-CoV-2 spike protein as well as neutralizing Abs after a period of 6 months in patients with the inflammatory joint disease [rheumatoid arthritis (RA) or spondyloarthritis (SpA)] and IBD as well as their response to a third (booster) vaccination.

## Materials and methods

### Patients

We enrolled patients with RA or seronegative SpA (including psoriatic arthritis, peripheral, and axial SpA) or ulcerative colitis and Crohn’s disease, who were followed up routinely at the outpatient clinics of the Department of Rheumatology and Gastroenterology of the Medical University of Vienna. This study is a follow-up of a cohort where we previously analyzed the response to the first two doses of SARS-CoV-2 mRNA vaccines (18). Now we focused on the antibody development 6 months after the first two vaccination doses and after the third immunization. All patients were vaccinated twice with an mRNA vaccine, and blood was taken 8 weeks and 5–6 months after the first vaccination (mean after 189 ± 22 days). Results and analysis of the antibody titers after 8 weeks were published in a previous article of our group (13). Patients with a history of SARS-CoV-2 infection were excluded. Patients receiving conventional synthetic disease-modifying antirheumatic drugs (csDMARDs) were compared with those receiving biological or targeted synthetic disease-modifying antirheumatic drugs (b/tsDMARDs) or a combination of csDMARDs and b/tsDMARDs. Healthy probands served as the control group (Table 1 and Supplementary Table 1).

**TABLE 1:** Characteristics of patients and controls.

		RA (*N* = 41)	PsA/SpA (*N* = 35)	IBD (*N* = 41)	HC (*N* = 85)
Age (years)		56.15 (±9.98)	50.69 (±13.22)	47.41 (± 11.67)	49.38 (±13.77)
Female		68.3% (*n* = 28)	51.4% (*n* = 18)	53.7% (*n* = 22)	60% (*n* = 51)
Male		31.7% (*n* = 13)	48.6% (*n* = 17)	46.3% (*n* = 19)	40% (*n* = 34)
**csDMARD**	**Methotrexate**	**24 (Mono *n* = 15)**	**9 (Mono *n* = 3)**	**1**	**None**
	Leflunomide	2 (Mono *n* = 1)	2		None
	Azathioprine	2 (Mono *n* = 2)		7 (Mono *n* = 3)	None
	Hydroxychloroquine	4 (Mono *n* = 2)			None
	Salazopyrin	1	1 (Mono *n* = 1)		None
	Mesalazine			18 (Mono *n* = 3)	
	Mycophenolate			1 (Mono *n* = 1)	
**bDMARD**					
TNF-inhibitor	Adalimumab		13 (Mono *n* = 8)	15 (Mono *n* = 12)	None
	Certolizumab		1 (Mono *n* = 1)		None
	Etanercept	2 (Mono *n* = 2)	2 (Mono *n* = 1)		None
	Golimumab	7 (Mono *n* = 2)	3 (Mono *n* = 3)	2 (Mono *n* = 1)	None
	Infliximab	2 (Mono *n* = 1)	1 (Mono *n* = 1)	5 (Mono *n* = 4)	None
IL-17 inhibitor	Secukinumab		4 (Mono *n* = 4)		None
	Ixekizumab		3 (Mono *n* = 2)		None
IL-6 inhibitor	Tocilizumab	3 (Mono *n* = 1)			None
IL-12/23 inhibitor	Ustekinumab			8 (Mono *n* = 6)	
Integrin-inhibitor	Vedolizumab			2	
**tsDMARD**					
JAK-inhibitor	Baricitinib	2 (Mono *n* = 1)			None
	Upadacitinib		1		None
	Filgotinib	1			None
Apremilast	Apremilast		2 (Mono *n* = 2)		None
No therapy		1	1	4	85
	Seropositive	*N* = 19			
Prednisolone dose	Patients without prednisolone	*N* = 34	*N* = 33	*N* = 40	*N* = 85
	Patients with daily prednisolone at 1. vaccination	*N* = 7 Mean dose: 7.7 mg/dl (±8.1)	*N* = 2 Mean dose: 6.2 mg/dl (±1.7)	*N* = 1 Mean dose: 5 mg/dl	None
	Patients without prednisolone	*N* = 34	*N* = 33	*N* = 40	*N* = 85
	Patients with daily prednisolone at 2. vaccination	*N* = 7 Mean dose: 7.7 mg/dl (±8.1)	*N* = 2 6.2 mg/dl (±1.7)	*N* = 1 Mean dose: 5 mg/dl	None

Age, CRP, and prednisolone dose are shown as mean (± SD).

Individuals without known immune-mediated inflammatory disease and no current intake of any immunomodulatory therapy including glucocorticoids, who were vaccinated twice with an mRNA vaccine, served as HC. HC with a history of SARS-CoV-2 infection was excluded. Blood was taken 8 weeks and 5–8 months (median 229 ± 32 days) after the first vaccination and 4 weeks after the third vaccination (median 31 days after the third vaccination for all groups). Ethical approval for this study was granted by the ethics committee of the Medical University of Vienna, Austria (1,291/2021; 559/2005; 1,073/2021). Patients and/or the public were not involved in the design, conduct, reporting, or dissemination plan of this research.

#### Anti-SARS-CoV-2 testing

Serum samples were stored at the Biobank of the Medical University of Vienna, a centralized facility for the preparation and storage of biomaterial with certified quality management (ISO 9001:2015) (19). The Elecsys^®^ Anti-SARS-CoV-2 S immunoassay was used for the quantitative determination of Abs to the receptor-binding domain (RBD) of the viral spike (S) protein (18). The quantitation range is between 0.4 and 2500.0 binding antibody units (BAU)/ml. Previous SARS-CoV-2 infection was assessed by measuring nucleocapsid-specific Abs with the qualitative Elecsys Anti-SARS-CoV-2 assay (20). Both tests were performed on a cobas^®^ e801 analyzer (Roche Diagnostics, Rotkreuz, Switzerland) at the Department of Laboratory Medicine, Medical University of Vienna (certified according to ISO 9001:2015 and accredited according to ISO 15189:2012).

### SARS-CoV-2 neutralization test (NT)

The NT was performed as described previously (21). Twofold serial dilutions of heat-inactivated serum samples were incubated with 50–100 tissue culture infectious dose 50% (TCID50) SARS-CoV-2 for 1 h at 37°C before the mixture was added to Vero E6 (ATCC CRL-1586) cell monolayers. Incubation was continued for 3 days. NT titers were expressed as the reciprocal of the serum dilution required for protection against virus-induced cytopathic effects. NT titer ≥ 10 was considered positive.

### Statistical analysis

Variables are depicted as the median and interquartile range (IQR) or mean and standard deviation (m ± SD), depending on their distribution. To investigate differences in anti-SARS-CoV-2 S protein titers between patients and HC, either Student’s *t*-test or one-way analysis of variance (ANOVA) or Kruskal–Wallis test was used, depending on the distribution, adjusting for multiple testing if necessary.

Under the assumption of a logarithmic decline in antibody levels over time, the time period above a threshold antibody level of 300 BAU was estimated for each patient. A Kruskal–Wallis test and subsequent Dunn’s test, adjusting for multiple testing using the Benjamini–Hochberg method, were performed to compare times above a threshold titer between HC and different treatment groups. Differences in the rate of decline in antibody levels over time were assessed by predicting slopes using a linear model adjusted for initial antibody levels. In addition, the difference and the relative difference between initial antibody levels and antibody levels after 6 months were compared between groups using a linear model adjusted for initial antibody levels and the time between the two antibody measurements. Antibody level decline was visualized through the estimation of mean logarithmic decline curves for each group by separately averaging over intercepts and slopes. Multivariable logistic regression analysis was used to predict an anti-SARS-CoV-2 S titer > 300 BAU/ml at the 6-month time point, with treatment [type of DMARD, glucocorticoids (yes/no), age, and time in days of anti-SARS-CoV-2 S titer measurement after the first immunization] as independent variables.

GraphPad Prism (version 9.1.0), IBM SPSS Statistics (version 26) as well as R (version 3.5.2) with Rstudio (version 1.4.1103) and the packages openxlsx, tidyr, dplyr, ggplot2, and ggbreak were used for statistical analysis and graphical presentation of the data.

## Results

**FIGURE 1:**
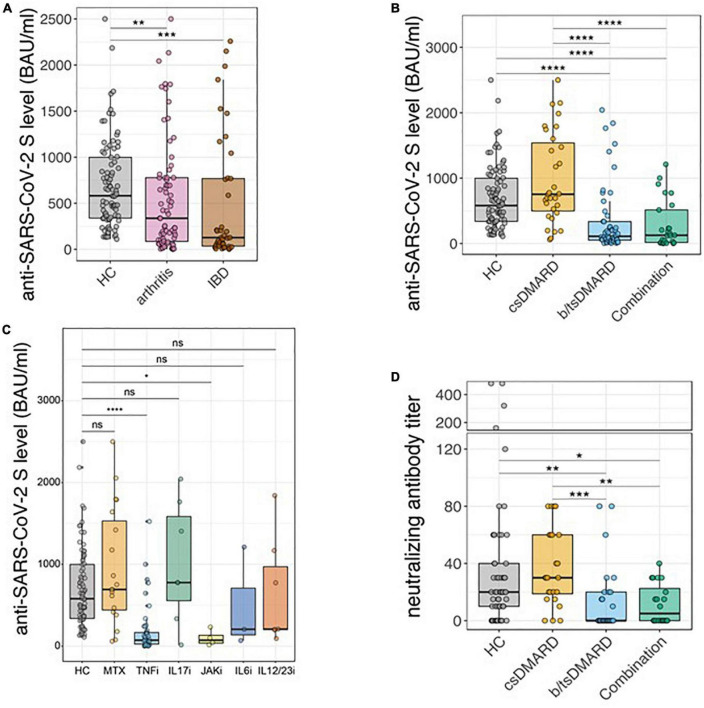
**(A)** Analysis of anti-SARS-CoV-2 S titers 6 months after the first two vaccination doses in HC (*n* = 85) and patients with inflammatory arthritis (*n* = 75) and inflammatory bowel disease (*n* = 41). **(B)** Analysis of anti-SARS-CoV-2 S titers 6 months after the first two vaccination doses comparing different treatment classes HC (*n* = 85), csDMARD (*n* = 30), b/tsDMARD (*n* = 54), and a combination of csDMARD and b/tsDMARD (*n* = 28). **(C)** Anti-SARS-CoV-2 S titers 6 months after the first two vaccination doses in patients with IBD and inflammatory arthritis according to the specific immunomodulatory treatments. HC (*n* = 85), methotrexate monotherapy (MTX mono; *n* = 19), tumor necrosis alpha-inhibitors (TNFi; *n* = 48), interleukin-17 inhibitors (IL-17i; *n* = 7), Janus kinase inhibitor (JAKi; *n* = 4), interleukin-6 receptor inhibitors (IL-6Ri; *n* = 3), interleukine-12/23 inhibitor (IL-12/23i *n* = 7). **(D)** Determination of neutralizing antibody activity in sera of HC (*n* = 56) and patients with inflammatory arthritis (*n* = 75) receiving the indicated therapies. Statistics used: Kruskal–Wallis test and subsequent Dunn’s test, adjusting for multiple testing using the Benjamini–Hochberg method. ns: non significant *p* > 0.05; *p ≤ 0.05; **p ≤ 0.01; ***p ≤ 0.005; and ****p ≤ 0.001.

We analyzed 75 patients with IA (RA and SpA), 41 patients with IBD, and 85 HC of which we had data of 6 months or more after the first vaccination. The demographic characteristics are shown in Table 1. Overall, we found reduced anti-SARS-CoV-2 S titers in patients with arthritis and IBD compared to the HC group [IA median 365 (IQR 91; 782), IBD median 128 (IQR 34; 840); HC median 581 (IQR 337; 1,003)] (Figure 1A). When we stratified our patients according to the class of immunomodulatory treatment (csDMARD monotherapy, b/tsDMARD monotherapy, csDMARD, and b/tsDMARD combination therapy), anti-SARS-CoV-2 S titers were significantly reduced in patients receiving both b/tsDMARD monotherapy [*n* = 54; median 111 (IQR 52; 359)] and a combination of csDMARDs and b/tsDMARDs [*n* = 28; median 168 (IQR 20; 571)] compared with HC [*n* = 85; median 581 (IQR 337; 1,003)]. Importantly, anti-SARS-CoV-2 S titers in patients receiving csDMARDs alone were similar to those of HC [*n* = 30; median 780 (IQR 579; 1,667); Figure 1B]. We observed an identical pattern when we analyzed the group of patients with IA and IBD separately (Supplementary Figure 1). In an exploratory analysis, after stratifying patients according to the most prevalent modes of action, anti-SARS-CoV-2 S titers at the 6-month time point were comparable in patients receiving methotrexate (MTX) compared with HC (Figure 1C). In contrast, anti-SARS-CoV-2 S titers were reduced in patients receiving tumor necrosis factor-α-inhibitor (TNFi) and Janus kinase inhibitor (JAKi), either alone or in combination with a csDMARD, compared to HC. Anti-SARS-CoV-2 S titers in patients receiving drugs blocking the IL-6 receptor and IL-12p40 were also reduced, but not in those receiving IL-17 blocking agents, although the number of patients in these groups was low (Figure 1C). We noted that the csDMARD group seemed to be split into two subgroups with higher and lower anti-SARS-CoV-2 S titers. We, therefore, analyzed whether there were significant differences in anti-SARS-CoV-2 S titers when we stratified these patients according to age (<57 years vs. > 57 years), diagnosis (RA vs. SpA), disease duration (0–5 years vs. > 5 years), and type of csDMARD (MTX vs. others). We found significantly lower anti-SARS-CoV-2 S titers in patients taking MTX compared with patients on csDMARDs other than MTX (i.e., azathioprine, hydroxychloroquine, and sulfasalazine) (Supplementary Figure 2).

Next, we investigated whether there is a difference in the presence of neutralizing Abs between the treatment groups in patients with IA. In line with the anti-SARS-CoV-2 S titers, no differences in neutralization capacity between patients with csDMARDs and HC were found. However, patients receiving b/tsDMARD monotherapy, either alone or in combination with a csDMARD, had significantly reduced titers of neutralizing Abs when compared with HC or patients receiving csDMARDs (Figure 1D). Importantly, while in HC and patients on csDMARDs, the proportion of people without detectable neutralizing Abs was 23 and 12%, respectively, this number being higher for patients on b/tsDMARDs (62%); 50% of patients receiving a combination of csDMARDs and b/tsDMARDs had no detectable neutralizing Abs.

**FIGURE 2:**
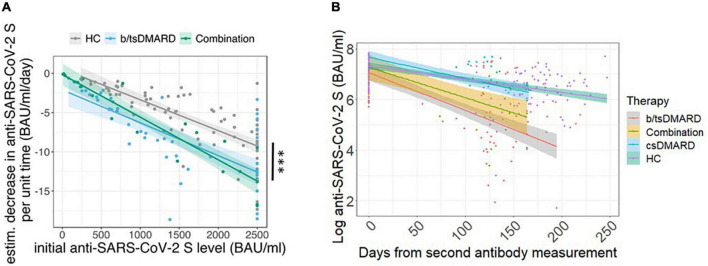
**(A)** Rate of decline in anti-SARS-CoV-2 S titers at a given peak antibody titer according to the indicated treatment: b/tsDMARDs (*n* = 54; light blue) and a combination of csDMARDs and b/tsDMARDs (*n* = 28; green) in patients vs. HC (*n* = 85; gray). Colored lines indicate linear regression. Thick colored lines indicate the mean slopes of the indicated patient group or HC. Indicated *p-*values are derived from a linear regression model with the estimated decrease (BAU/ml/day) as predicted variable and initial anti-SARS-CoV-2 S titers, therapy, and age as independent variables. Statistics used: Kruskal–Wallis test and subsequent Dunn’s test. **(B)** Decline of anti-SARS-CoV-2 S titers from peak levels after the second immunization and the 6-month time point in HC (*n* = 85; green) and patients receiving csDMARDs (*n* = 23; blue), b/tsDMARDs (*n* = 47; gray), and a combination of csDMARDs and b/tsDMARDs (*n* = 14; yellow). Thick colored lines indicate the mean slopes of the indicated patient group or HC. ns: non significant *p* > 0.05; *p ≤ 0.05; **p ≤ 0.01; ***p ≤ 0.005; and ****p ≤ 0.001.

The waning of the anti-SARS-CoV-2 S titers was reported to be logarithmic (22). Assuming a logarithmic rate of decrease, we found that the rate of decline was dependent on initial peak antibody levels 3–4 weeks after the second vaccination in both patients and HC. However, this decline was significantly accelerated at any given peak antibody level in patients (in both IA and IBD) receiving b/tsDMARD, either as monotherapy or in combination with csDMARDs, but not in patients receiving only csDMARDs compared to HC (Figure 2A and Supplementary Figure 3). In addition, we also analyzed the decline of anti-SARS-CoV-2 S titers over time. We found a steeper decline of anti-SARS-CoV-2 S titers after the second vaccination in patients receiving b/tsDMARDs either alone or in combination with csDMARDs compared to HC and patients on csDMARDs alone (Figure 2B).

**FIGURE 3:**
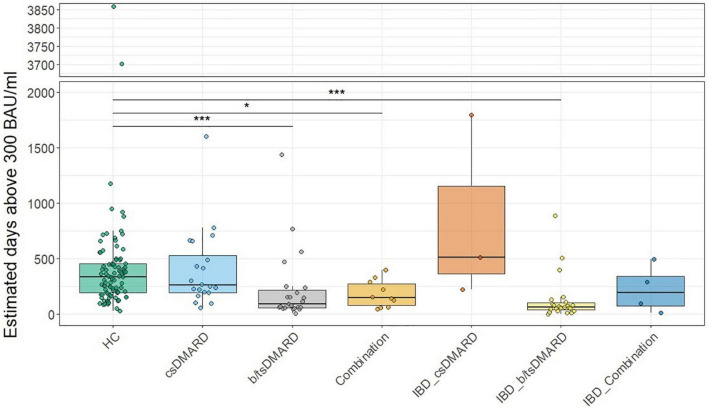
Estimation of median days above 300 BAU/ml in HC or inflammatory arthritis patients or inflammatory bowel disease (IBD) patients receiving the indicated treatments: csDMARD (*n* = 30), b/tsDMARD (*n* = 54), and a combination of csDMARD and b/tsDMARD (*n* = 28) compared with healthy controls. Statistics used: Kruskal–Wallis test and subsequent Dunn’s test. ns: non significant *p* > 0.05; *p ≤ 0.05; **p ≤ 0.01; ***p ≤ 0.005; and ****p ≤ 0.001.

We then calculated the number of days patients or HC remained above a certain threshold of anti-SARS-CoV-2 S titers following the peak antibody response 3–4 weeks after the second vaccination. We chose a threshold of 300 BAU/ml, as we did not observe the presence of neutralizing Abs below that threshold in our measurements (data not shown). In both IA and IBD, the estimated median number of days above the 300 BAU/ml threshold was significantly lower in patients receiving b/tsDMARDs, either alone or in combination with a csDMARD, than in patients receiving csDMARDs or in HC (Figure 3). In HC and patients receiving csDMARDs, the percentage of patients with a titer above 300 BAU/ml was around 80% at the 6-month time point. This percentage was markedly lower in patients receiving b/tsDMARDs either alone or in combination with csDMARDs (35 and 30% in patients receiving b/tsDMARDs either alone or in combination with csDMARDs and in IA patients, 12% in IBD patients receiving b/tsDMARDs) (Supplementary Figure 4). In multivariate logistic regression models, odds ratios (OR) predicting an anti-SARS-CoV-2 S titer above 300 BAU/ml were significantly higher in those starting with a high initial titer, but lower for patients receiving b/tsDMARDs, combination therapy, glucocorticoids (yes/no) at baseline, and age compared to HC (Supplementary Figure 5). Therefore, treatment with b/tsDMARD, either as monotherapy or in combination with csDMARDs, was associated with a reduction in the duration of vaccine-induced humoral immunity by almost 50%. These data demonstrate an accelerated decline of anti-SARS-CoV-2 S titers in patients receiving b/tsDMARDs and, therefore, a reduced duration of vaccination-induced immunity.

**FIGURE 4:**
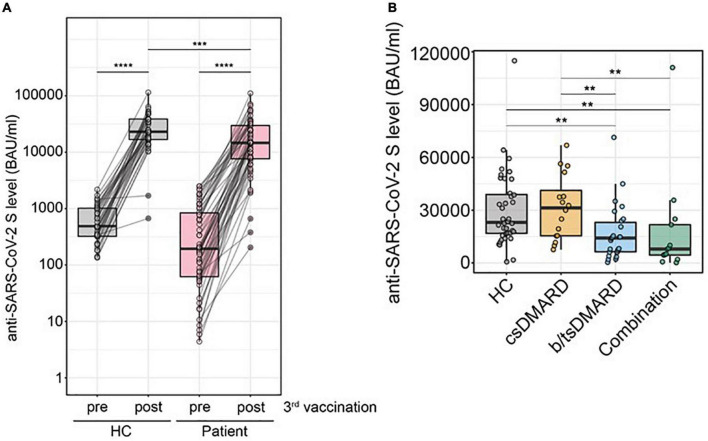
**(A)** Comparison of anti-SARS-CoV-2 S antibody levels before and after a third immunization with an mRNA vaccination in HC (*n* = 40) vs. patients (*n* = 58) (Mann–Whitney *U*-test). **(B)** Anti-SARS-CoV-2 S antibody levels after the third immunization of patients with Inflammatory arthritis (*n* = 38) and inflammatory bowel disease (IBD; *n* = 20) receiving csDMARDs (*n* = 16), b/tsDMARDs (*n* = 26), or a combination of both (*n* = 13). Statistics used: Kruskal–Wallis test and subsequent Dunn’s test. ns: non significant *p* > 0.05; *p ≤ 0.05; **p ≤ 0.01; ***p ≤ 0.005; and ****p ≤ 0.001.

We next determined the effect of a third (booster) vaccination in a subgroup of our patients. Anti-SARS-CoV-2 S titers significantly increased in all patients as well as HC, with a median fold increase of 63 in the patient group and 50 in HC, this difference being not statistically significant (Figure 4A). However, anti-SARS-CoV-2 S antibody levels were significantly lower in the patient group compared to HC after booster vaccination (Figure 4A). There was no difference in anti-SARS-CoV-2 S antibody levels between HC [*n* = 40; median 23,085 (IQR 16,745; 39,375)] and patients with IA [*n* = 38; median 14,980 (IQR 7,763; 34,650)]. However, we detected reduced anti-SARS-CoV-2 S antibody levels in patients with IBD [*n* = 20; median 13,045 (IQR 6,323; 24,723), *p* < 0.05] compared with HC. When we analyzed anti-SARS-CoV-2 S antibody levels after the third immunization stratified for the therapies, we found that patients receiving csDMARDs (*n* = 16) were not different from HC (Figure 4B). However, both patients with b/tsDMARD monotherapy (*n* = 26) and patients with combination therapy of csDMARD and b/tsDMARD (*n* = 13) showed reduced anti-SARS-CoV-2 S levels compared with HC (Figure 4B). When we analyzed distinct modes of action (MOA), we found that anti-SARS-CoV-2 S antibody levels after the third immunization of patients receiving MTX [*n* = 12 median 24,675 (IQR 12,523; 48,075)] were not different from HC [*n* = 40 median 23,085 (IQR 16,745; 39,375)]. In contrast, patients receiving TNFi [*n* = 28; median 8,305 (IQR 4,430; 15,260), *p* < 0.001] showed significantly reduced anti-SARS-CoV-2 S antibody levels after the third vaccination compared to HC, with other MOAs being not prevalent enough to allow meaningful analysis. We then performed linear regression models to predict anti-SARS-CoV-2 S antibody levels after the third vaccination, with age, disease status, anti-SARS-CoV-2 S antibody titers at the 6-month time point, and its interaction with disease status (HC vs. patients) as independent variables. These models suggested an effect of anti-SARS-CoV-2 S levels at the 6-month time point in predicting response to the third vaccination in patients for all forms of treatment in patients, but not in HC, with possibly the largest effect in patients on combination therapies (Supplementary Tables 2–4). These results suggest that the rapid decline of anti-SARS-CoV-2 S antibody titers in patients receiving DMARD therapy is associated with a reduced response to the booster vaccination.

## Discussion

In our study, we found that patients receiving certain immunomodulatory therapies have a significantly accelerated decline of protective immunity after immunization against SARS-CoV-2. We have recently found that peak immune responses to SARS-CoV-2 vaccination were largely similar between patients with IA receiving immunomodulatory treatments and HC (18). In this study, we analyzed antibody development after a follow-up period of 6 months in the same patient cohort as well as the response of a subgroup of these patients to a third vaccination. We found that b/tsDMARDs significantly reduce the duration of vaccine-induced protection in patients with both IA and IBD. These findings go hand in hand with various studies focusing on IBD or IA, yet not comparing these rheumatic and other autoinflammatory diseases (e.g., IBD) with respect to therapy (23, 24). This reduced duration of the vaccine response was independent of underlying immune-mediated disease but clearly associated with the type of immunomodulation. These data highlight the need for individualized vaccination strategies in order to maximize the prevention of disease in those patients and provide a rationale for vaccination recommendations in patients treated with immunomodulatory drugs. As patients receiving b/tsDMARD or combination therapy display lower antibody levels, these patients may benefit from an earlier booster vaccination. In addition, we would argue that anti-SARS-CoV-2 antibody levels should be measured in patients receiving DMARDs to allow earlier booster vaccination for a third and maybe even a fourth time. While a specific cutoff value for protective immunity has not been established yet, in our study, we chose 300 BAU/ml of anti-SARS-CoV-2 S titers, as in our cohort we did not observe neutralizing antibody activity below that value. A limitation of our study is that we only measured antibody levels after 6 months, which was specified in our protocol at the beginning of the study.

Our study is in line with recent studies also noting a gradual waning of anti-SARS-Cov-2 antibody titers in HC as well as in patients with autoimmune diseases (22, 25). In our analysis, we excluded patients receiving B-cell-depleting therapies, who were reported to have significantly reduced initial responses to SARS-CoV-2 immunization (12, 26). Nonetheless, it will be important to determine the impact of B-cell-depleting therapies on existing anti-SARS-CoV-2 S titers.

Although we do have a mixture of different therapies present in our b/tsDMARD group, we found that the analysis of the most prevalent MOA, namely, TNFi, was similar to the whole group of bDMARDs. This allows us to conclude that, although peak antibody levels measured about 1 month after the second mRNA vaccination were equivalent to those of HC, TNF inhibition was associated with an accelerated decline of anti-spike-IgG antibodies and neutralization capacity in patients with immune-mediated inflammatory diseases. It will be interesting to analyze the impact of TNFi on other immune-mediated diseases such as psoriasis, where this class of DMARDs is widely used, as well as other classes of immunomodulatory drugs in this regard. Interestingly, one report did not find differences in anti-SARS-CoV-2 S titers in patients with psoriasis treated with TNFi, suggesting that both the type of disease and treatment might affect outcomes (27).

Our data suggest that csDMARDs do not affect the duration of the humoral immune response after SARS-CoV-2 vaccination, whereas b/tsDMARD therapy clearly does so. This is in line with some other reports (11, 28), while others did find an influence of csDMARDs, especially MTX, in the humoral immune response after SARS-Cov-2 vaccination (10, 29). These discrepancies are possibly due to the time of analysis and age differences in the populations studied (30). In light of these data, it will be necessary to monitor the immune response in patients receiving certain immunomodulatory drugs even after booster vaccination. The implication of this observation goes beyond SARS-CoV-2, as the impact of b/tsDMARDs possibly also affects the duration of humoral immune responses of other vaccinations.

This faster decline in anti-SARS-CoV-2 S titers was also associated with a reduced anti-SARS-CoV-2 titer development after a third immunization (booster vaccination). While the relative increase in anti-SARS-CoV-2 titers was even higher in patients compared with HC, this suggests that the accelerated decline and, therefore, lower anti-SARS-CoV-2 levels before the third vaccination were responsible for this observation.

## Conclusion

We demonstrate a reduced duration of protective humoral immune responses in patients with IA and IBD receiving b/tsDMARD therapy after two doses of an mRNA vaccine against SARS-CoV-2, which was even associated with lower Abs levels after booster vaccination. These data form the basis for personalized vaccination strategies that are urgently needed to maximize vaccine-mediated protection against SARS-CoV-2 in a vulnerable group of patients.

## Data availability statement

The original contributions presented in this study are included in the article/Supplementary material, further inquiries can be directed to the corresponding author.

## Ethics statement

The studies involving human participants were reviewed and approved by the Ethics Committee of the Medical University of Vienna, Austria (1291/2021, 559/2005, and 1073/2021). The patients/participants provided their written informed consent to participate in this study.

## Author contributions

ES, ST, HB, KS, SW, DA, and SB designed the study. ST, PM, TD, TN, LS, FW, SB, ES, HH, KS, HR, TP, JS, FK, and LH analyzed the data. ST, PM, TD, LS, ES, HH, TP, HB, DA, SW, SB, JS, FK, and LH interpreted the results. ST, ES, PM, DA, and SB wrote the manuscript. All authors revised the manuscript and were involved in editing and quality control.
