# Supplementary material for: The accelerated waning of immunity and reduced effect of booster in patients treated with bDMARD and tsDMARD after SARS-CoV-2 mRNA vaccination
Source: Front Med (Lausanne). 2023 Feb 9;10:1049157. doi: 10.3389/fmed.2023.1049157 (PMC9947701; doi:10.3389/fmed.2023.1049157)
Supplement: Supplementary file 1 [file Data_Sheet_1.PDF]

Supplementary Figure 1

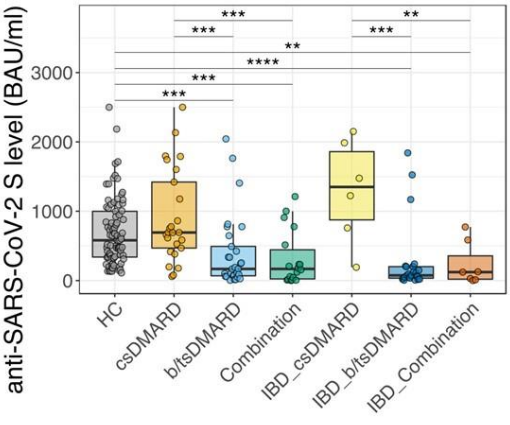

Suppl. Fig. 1

Analysis of anti-SARS-CoV-2 S titers 6 months after the first two vaccination doses in HC (n=85), patients with inflammatory arthritis receiving csDMARD (n=24), b/tsDMARD (n=29), combination of csDMARDs and b/tsDMARD (n=29), or IBD patients receiving csDMARD (n=6), b/tsDMARD (n=25), combination of csDMARDs and b/tsDMARD (n=7) (* $p \leq 0.05$; **** $p \leq 0.001$; Statistics used: Kruskal-Wallis test and subsequent Dunn’s test.

Supplementary Figure 2

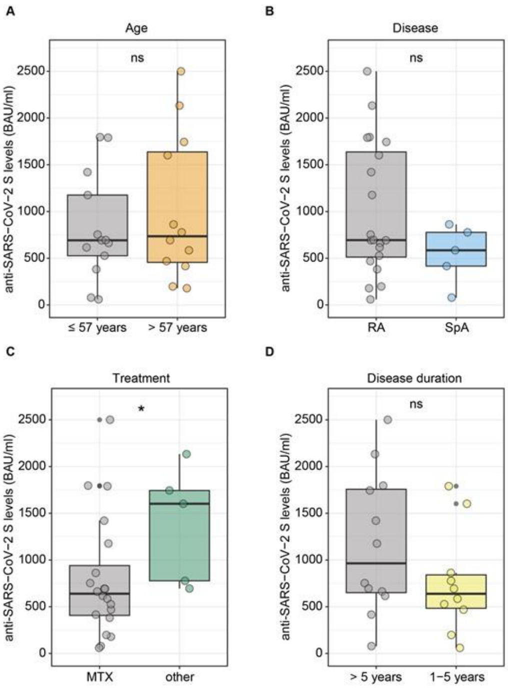

Suppl. Fig. 2

Comparison of anti-SARS-CoV-2 S antibody levels 6 months after the first two vaccination doses in patients receiving csDMARDs according to **A,** age > 57 years; n=13 vs. < 57 years; n=12; **B,** diagnosis (RA n=20 vs. SpA n=5) **C,** disease duration <5 years (n=10) vs. > 5 years (n=12), **D,** medication (MTX, n=20) vs. other csDMARDs (azathioprine, hydroxychloroquine or sulfasalazine) (n=5). (* $p \leq 0.05$; Statistics used: Unpaired t test or Mann-Whitney U test according to normality test.

Supplementary Figure 3

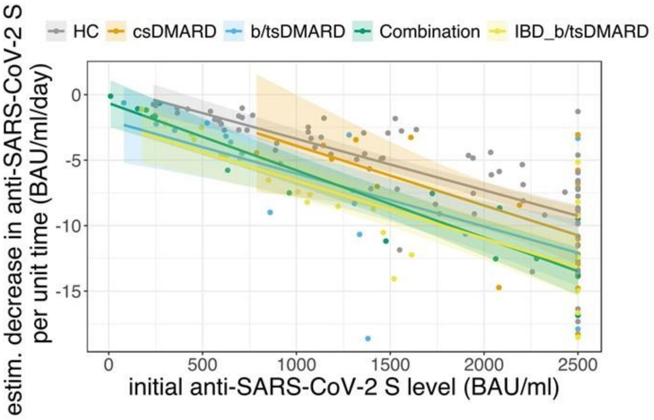

Suppl. Fig. 3

Rate of decline in anti-SARS-CoV-2 S titers at a given peak antibody titer according to the indicated treatment in patients with inflammatory arthritis (csDMARDs (orange), b/tsDMARDs (light blue) and a combination of and csDMARDs and b/tsDMARDs (green) as well as patients with inflammatory bowel disease treated with b/tsDMARDs (yellow). Colored line indicates linear regression. Indicated p values are derived from a linear regression model with the estimated decrease (BAU/ml/day) as predicted variable and initial anti-SARS-CoV-2 S titers, therapy and age as independent variables. Statistics used: Kruskal-Wallis test and subsequent Dunn's test.

Supplementary Figure 4

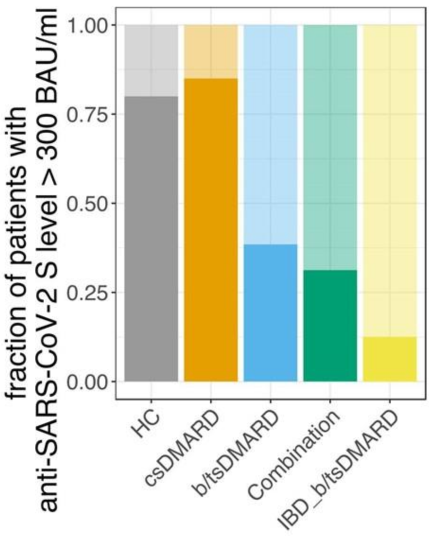

Suppl. Fig. 4

Fraction of HC (n=85), patients with inflammatory arthritis (n=75) and inflammatory bowel disease (n=41) with antibody levels > 300 BAU/ml receiving the indicated treatment 6 months after after the first two vaccination doses.

Supplementary Figure 5

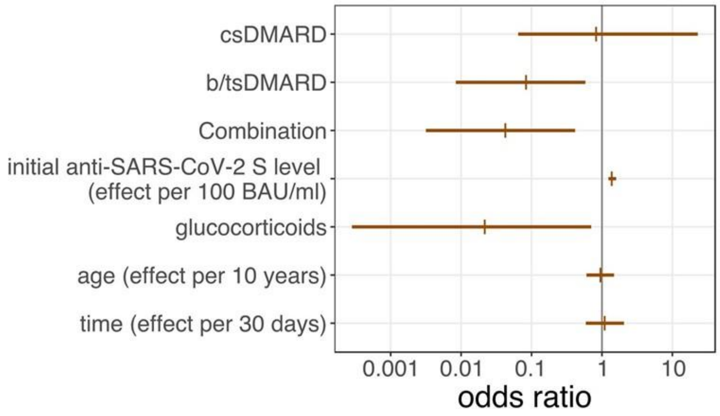

Suppl. Fig. 5

Odds ratios of multivariate logistic regression predicting an anti-SARS-CoV-2 S titer above 300 BAU/ml 6 months after the first two vaccination doses. csDMARDs were used as reference category Statistics used: Multivariable logistic regression analysis.
