# Supplementary material for: The accelerated waning of immunity and reduced effect of booster in patients treated with bDMARD and tsDMARD after SARS-CoV-2 mRNA vaccination
Source: Front Med (Lausanne). 2023 Feb 9;10:1049157. doi: 10.3389/fmed.2023.1049157 (PMC9947701; doi:10.3389/fmed.2023.1049157)
Supplement: Supplementary file 2 [file Data_Sheet_2.pdf]

**Supplementary table 1:** **Characteristics of patients and controls receiving a third vaccination. Age, CRP and prednisolone dose are shown as mean (± SD).**

| | | RA (N=18) | PsA/SpA (N=19) | IBD (N=20) | HC (N=40) |
|---|---|---|---|---|---|
| **Age (years)** | | 57,2 (±9,3) | 49,7 (±13,6) | 45,6 (±10,7) | 51,7 (±17,2) |
| **Female** | | 77,8% (n=14) | 63,2% (n=12) | 65 % (n=13) | 57,5% (n=23) |
| **Male** | | 22,2% (n=4) | 36,8% (n=7) | 35% (n=7) | 42,5% (n=17) |
| ***csDMARD*** | | | | | |
| | Methotrexat | 12 (Mono n=9) | 6 (Mono n=3) | 1 (Mono n=0) | none |
| | Leflunomid | 1 (Mono n=0) | 1 (Mono n=0) | | none |
| | Azathioprin | none | | 2 (Mono n=1) | none |
| | Hydroxycholorquin | 2 (Mono n=1) | | | none |
| | Salazopyrin | 1 (Mono n=0) | 1 (Mono n=1) | | none |
| | Mesalazin | | | 2 (Mono n=1) | |
| | Mycophenolat | | | 1 (Mono n=1) | |
| ***bDMARD*** | | | | | |
| **TNF-Inhibitor** | Adalimumab | | 8 (Mono n=6) | 7 (Mono n=2) | none |
| | Certolizumab | | 1 (Mono n=1) | | none |
| | Etanercept | 1 (Mono n=1) | 1 (Mono n=0) | | none |
| | Golimumab | 4 (Mono n=0) | 1 (Mono n=1) | 2 (Mono n=1) | none |
| | Infliximab | 2 (Mono n=1) | | 2 (Mono n=2) | none |
| **IL-17 Inhibitor** | Secukinumab | | 3 (Mono n=3) | | none |
| | Ixekizumab | | | | none |
| **IL-6 Inhibitor** | Tocilizumab | 2 (Mono n=0) | | | none |
| **IL-12/23 Inhibitor** | Ustekinumab | | | 3 (Mono n=3) | |
| **Integrin-Inhibitor** | Vedolizumab | | | | |
| ***tsDMARD*** | | | | | |
| **JAK-Inhibitor** | Baricitinib | | | | none |
| | Upadacitinib | | 1 (Mono n=0) | | none |
| | Filgotinib | | | | none |
| **Apremilast** | Apremilast | | | | none |
| **Integrin-Inhibitor** | Vedolizumab | | | | |

| | | | | | |
|---|---|---|---|---|---|
| ***No therapy*** | | | | 3 | 40 |
| | Seropositive | N=6 | | | |
| **Prednisolone dose** | Patients without prednisolone | N=16 | N= 19 | N= 20 | N= 40 |
| | Patients with daily prednisolone at 1. vaccination | N=2<br>Mean dose: 7,5mg/dL(±7,1) | N=0<br>Mean dose: 0mg/dL | N=1<br>Mean dose: 5mg/dL | none |
| | Patients without prednisolone | N= 15 | N= 18 | N= 20 | N= 40 |
| | Patients with daily prednisolone at 2. vaccination | N=3<br>Mean dose: 9,2mg/dL(±9,4) | N=1<br>25mg/dL | N=1<br>Mean dose: 5mg/dL | none |
| | | | | | |

**Supplementary table 2:** **Multivariable regression to predict anti-SARS-CoV-2 S antibody-level after third vaccination for patients**

| Variable | Levels | coefficient | 95% CI | | p-value |
|---|---|---|---|---|---|
| | | | LL | UL | |
| **Age** | | 25.345 | -384.944 | 435.634 | 0.901 |
| **Antibody level at 6 month time point (effect per 1 BAU/ml)** | | 12.884 | 62.771 | 25.141 | 0.0397 * |
| **Therapy** | csDMARD | reference | reference | | |
| | b/tsDMARD | -7212.017 | -24419.43 | 9995.399 | 0.403 |
| | combination | -13012.07 | -31751.41 | 5727.266 | 0.169 |
| | | | | | |
| **Interaction disease status/antibody level at 6 month time point** | | | | | |
| | csDMARD | reference | reference | | |
| | b/tsDMARD | 1.671 | -14.409 | 17.750 | 0.835 |
| | combination | 38.064 | 10.090 | 66.038 | 0.009 ** |

Abbreviation: CI Confidence interval, LL lower limit; UL upper limit

**Supplementary table 3: Multivariable regression to predict anti-SARS-CoV-2 S antibody-level after third vaccination**

| Variable | Levels | coefficient | 95% CI | | p-value |
|---|---|---|---|---|---|
| | | | LL | UL | |
| **Age** | | 117.884 | -166.220 | 401.989 | 0.4119 |
| **Antibody level at 6 month time point (effect per 1 BAU/ml)** | | 1.102 | -11.715 | 13.920 | 0.8647 |
| **Disease status** | Healthy | reference | reference | | |
| | Patient | -14774.11 | -26293.956 | -3254.27 | 0.0125 * |
| **Interaction disease status/antibody level at 6 month time point** | | | | | |
| | Healthy | reference | reference | | |
| | Patient | 16.965 | 2.634 | 31.296 | 0.0209 * |

Abbreviation: CI Confidence interval, LL lower limit; UL upper limit;

**Supplementary table 4: Multivariable regression to predict anti-SARS-CoV-2 S antibody-level after third vaccination for healthy controls**

| Variable | Levels | coefficient | 95% CI | | p-value |
|---|---|---|---|---|---|
| | | | LL | UL | |
| **Age** | | 121.547 | -291.471 | 534.564 | 0.554 |
| **Antibody level at 6 month time point (effect per 1 BAU/ml)** | | 1.147 | -11.939 | 14.232 | 0.860 |

Abbreviation: CI Confidence interval, LL lower limit; UL upper limit
